# Effect of Nusinersen in a late onset spinal muscular atrophy patient for 14 months: A case report

**DOI:** 10.1097/MD.0000000000024236

**Published:** 2021-01-08

**Authors:** Jin-Mo Park, Yu-Sun Min, Donghwi Park, Jin-Sung Park

**Affiliations:** a Department of Neurology, Dongguk University College of Medicine, Dongguk University Gyeongju Hospital, Gyeongju; b Department of Rehabilitation, Kyungpook National University Chilgok hospital, School of Medicine, Kyungpook National University, Daegu; c Department of Physical Medicine and Rehabilitation, Ulsan University Hospital, University of Ulsan College of Medicine; d Department of Neurology, Kyungpook National University Chilgok Hospital, School of Medicine, Kyungpook National University, Daegu, South Korea.

**Keywords:** 6-minute walk test, adverse event, Hammersmith rating scale, Nusinersen, spinal muscular atrophy

## Abstract

**Rationale:**

Spinal muscular atrophy (SMA) is a genetic disorder caused by genetic defect of *SMN1* gene. SMA was an untreatable disease until 2016, when Nusinersen an antisense oligonucleotide therapy was approved for treatment. We report the effect of Nusinersen in a late onset SMA for 14 months.

**Patient concerns:**

A 13-year-old boy who was diagnosed as SMA with progressive proximal limb weakness was treated with intrathecal injection of Nusinersen.

**Diagnosis:**

The patient had progressive proximal limb weakness after 2 years of age. The patient had elevated creatine kinase level and shoed neurogenic changes in the needle electromyography study. After genetic analysis, homozygous deletion in Exon 7 and 8 of SMN1 protein was found and he was diagnosed as late onset SMA.

**Interventions:**

Intrathecal Nusinersen was administered per protocol.

**Outcomes:**

After 14 months of treatment, the patient showed significant clinical improvement in the revised Hammersmith functional rating scale and 6-minute walk test.

**Lessons:**

Although there is limited data on the effect of Nusinersen in late onset SMA patients, our case adds on the effectiveness even in late onset SMA. More studies are needed to consolidate the effects and adverse events of Nusinersen in late onset SMA.

## 1 Introduction

Spinal muscular atrophy (SMA) is an autosomal recessive neuromuscular genetic disorder. It is caused by homozygous mutations in the survival motor neuron (*SMN*) 1 gene that leads to deficiency of SMN protein, which is predominant in the anterior horn cells.^[1]^ Recently, Nusinersen, an antisense oligonucleotide that modulates pre-mRNA splicing to promote exon 7 inclusion of the SMN2 mRNA transcripts resulting in enhanced production of a functional SMN protein was approved as a disease-modifying therapy for SMA. This drug is currently approved and used in the United States, Europe and Japan. The National Health Insurance Service of South Korea has also approved the use since April of 2019. Here, we report the first Korean case of late onset SMA treated with Nusinersen for 14 months.

## 2 Case presentation

**Figure 1:**
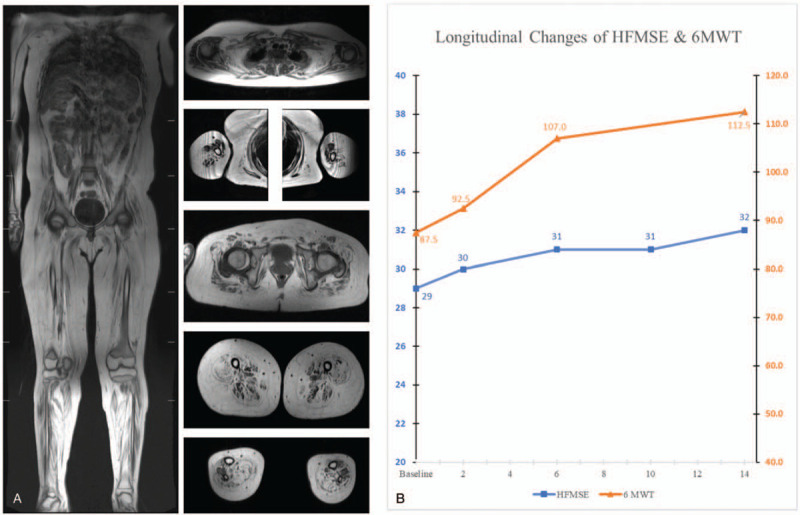
(A) Muscle MRI showed diffuse fatty change in the lower limb muscles with relative spared muscle involvement in the upper extremities (B) Longitudinal changes of HFMSE scores (0–66) and 6MWT (m) during 14 months treatment of Nusinersen.

A 13-year-old male patient was referred to our Neurology clinic due to progressive weakness. He was born by caesarian section and he had a delay in the milestone where he could stand by himself at 18 months. At the age of 3, muscle biopsy and genetic analysis of a homozygous deletion in Exon7-8 of *SMN1* led to diagnosis of SMA. The initial neurological examination showed proximal leg weakness of medical research council scale (MRC) of 2 and shoulder elevation weakness of MRC grade 3. He showed hypoactive deep tendon reflexes and a positive Gower sign. The laboratory findings were unremarkable except for a mild elevation of creatine kinase (312 um/L). We further proceeded with the genetic study via multiplex ligation dependent probe amplification and 3 copy number variation (CNV) of *SMN2* was detected. The nerve conduction study (NCS) was unremarkable, except for a reduced compound muscle action potential (CMAP) of tibial nerve and the needle electromyography showed giant motor unit potentials in all the tested muscles. The muscle magnetic resonance imaging (MRI) significant fatty infiltrations in the proximal and axial muscles (Fig. 1A). The patient was 15 when Nusinersen was approved in South Korea. The baseline Hammersmith functional motor scale extended (HFMSE) was 29 points, Medical Research Council sum score (MRCSS) was 42 and 6-minute walk test (6MWT) was 87.5 m before the treatment. The pulmonary function showed a forced vital capacity (FVC) of 4.65 L (114%), forced expiratory volume in one second (FEV1) of 3.55 L (103%) and FEV1/FVC of 89% which were within normal limits. Nusinersen was administered as protocol.^[2]^

After 14 months of treatment, the distance of 6MWT was changed from 87.5 to 113 m. The baseline CMAP before the treatment was 5.1 and 4.4 mV in both common peroneal nerves that changed to 5.9 and 4.6 mV after treatment. The follow-up pulmonary function test showed improvement of FVC of 129% (from 114%) and FEV1 of 110% (from 103%). The baseline Hammersmith functional motor scale extended (HFMSE) was 29 that changed to 32 (Fig. 1B). The item 13 of HFMSE, for ability to prop on extended arms, reflects the upper motor function and improved from score 1 to 2. The item 15 that checks whether the patient can complete a four-point kneeling improved also from 1 to 2.

## 3 Discussion

Nusinersen showed an improved motor function in phase III randomized controlled trials that lead to the drug approval in early onset SMA,^[2,3]^ but evidence of favorable outcome of Nusinersen in older patients are insufficient and there is a need to investigate on the effectiveness of late onset SMA as well. In accordance with this issue, the importance of this case is that it provides a clinical evidence in the effectiveness of Nusinersen in late onset SMA. The efficacy of Nusinersen in late SMA seems to be influenced by age.^[4]^ In this study, there was significant improvement of HFMSE but the degree of improvement in SMA type 3 was less than that of SMA type 2 (+1.8 points vs +10.8 points) showing a modest efficacy of Nusinersen in SMA type 3. This can be easily understood by the natural history data of growth in SMA where there is significant worsening of symptoms during adolescence where SMA children between 5 and 15 years were more likely to experience weight gain and increased contractures and scoliosis and showed the largest numerical change of deterioration.^[5,6]^ A recent observational cohort study was performed in 139 late onset SMA with a age ranging from 16 to 65 years old patients.^[7]^ They evaluated the treatment outcomes based on HFMSE at baseline, 6, 10, and 14 months. There was steady improvement of HFMSE from 1.73, 2.58, and 3.12. It is noteworthy to state that in this study patients who showed more than 10-point improvement were SMA type 3 with an age ranging from 48 to 59 years, reflecting the effect of Nusinersen regardless of age. In accordance with this study, the baseline HFMSE score of our case was 32 and achieved an improved score of 2 points after 14 months. Adverse events related to Nusinersen includes headache, back pain, nausea, constipation and dizziness in the descending order were reported in 82% of the patients.^[8]^ Our patient experienced no significant adverse event but had a mild headache only after the first injection that subsided with symptomatic treatment and there were no adverse events that prevented the patient from receiving Nusinersen.

## 4 Conclusion

In conclusion, our case showed the safety and efficacy of Nusinersen in late onset SMA patients without serious adverse events and to the best of our knowledge this is the first reported case of Korean SMA patients treated with Nusinersen. More studies on late onset SMA is needed to accurately understand the efficacy of Nusinersen and our case adds to the favorable outcome in the categories of late onset SMA patients.

## Author contributions

**Conceptualization:** Jin-Sung Park.

**Formal analysis:** Donghwi Park.

**Funding acquisition:** Jin-Mo Park.

**Investigation:** Jin-Mo Park.

**Methodology:** Jin-Mo Park, Yu-Sun Min, Donghwi Park.

**Writing – original draft:** Jin-Mo Park.

**Writing – review & editing:** Jin-Sung Park.
